# Helminth communities of endemic cyprinoids of the Apennine Peninsula, with remarks on ectoparasitic monogeneans, and a description of four new *Dactylogyrus* Diesing, 1850 species

**DOI:** 10.1017/S0031182021000615

**Published:** 2021-07

**Authors:** Michal Benovics, Kateřina Francová, Pietro Volta, Vojtěch Dlapka, Andrea Šimková

**Affiliations:** 1 Department of Botany and Zoology, Faculty of Science, Masaryk University, Kotlářská 2, 611 37, Brno, Czech Republic; 2 CNR IRSA Water Research Institute Verbania, Viale Tonolli 50, 289 22, Verbania, Italy

**Keywords:** Cyprinoidei, endemic species, Italian Peninsula, parasite diversity, phylogeny, taxonomy

## Abstract

The fauna of the Apennine Peninsula is, in comparison to other southern European peninsulas, relatively species-poor regarding the number of endemic cyprinoid species. Nonetheless, the recent introduction of non-native species has significantly increased the total number of freshwater species in this region. Such invasive species may represent a threat to the native fauna, associated among other things with the introduction of non-native parasites with their original hosts.

In the present study, we investigated endemic cyprinoid species for the presence of helminth parasites. A total of 36 ectoparasitic monogenean species and five endoparasitic helminth species were collected from ten cyprinoid species in five localities in northern Italy. Out of 20 *Dactylogyrus* species (gill monogeneans specific to cyprinoids), four were identified as new to science and herein described: *Dactylogyrus opertus* n. sp. and *Dactylogyrus sagittarius* n. sp. from *Telestes muticellus*, *Dactylogyrus conchatus* n. sp. from *T. muticellus* and *Protochondrostoma genei*, and *Dactylogyrus globulatus* n. sp. from *Chondrostoma soetta*. All new *Dactylogyrus* species appear to be endemic to the Apennine Peninsula; however, they share a common evolutionary history with the endemic *Dactylogyrus* parasitizing cyprinoids of the Balkans. This common origin of cyprinoid-specific parasites supports a historical connection between these two (currently separated) geographical regions.

## Graphical abstract

**Figure:**
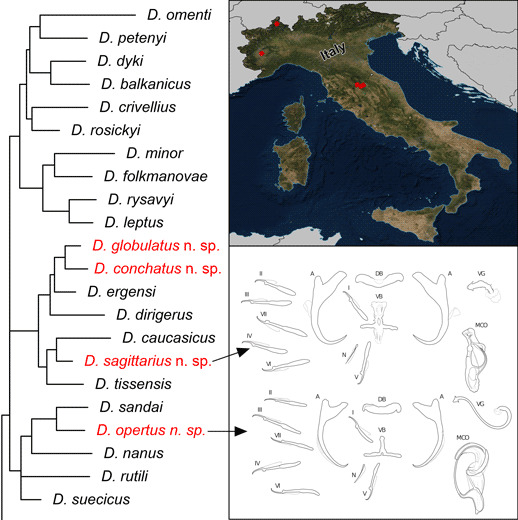

## Introduction

The composition of a parasite community is the result of multiple factors influencing parasitic organisms and their hosts (Poulin, 2007). Besides environmental factors (Galaktionov and Bustnes, 1999; Marcogliese, 2001; Maestri *et al*., 2017; Clark *et al*., 2018), the biology of a given host usually has a great impact on the species richness of parasite communities and the abundance of individual parasite species (or in combination with the environment, Berkhout *et al*., 2020). In general, the species richness of parasites is intimately linked with the species diversity of their hosts (Krasnov *et al*., 2004; Hechinger and Lafferty, 2005; Thieltges *et al*., 2011; Kamiya *et al*., 2014); however, both the range and spatial pattern of distribution of a given host species may affect parasite assemblages, as the resource availability varies across the whole distribution range of the host (Clark *et al*., 2018; Berkhout *et al*., 2020). Studying parasite communities and the phylogenetic relationships between parasite species (especially parasite species exhibiting high host-specificity) can shed light on the evolutionary history and historical dispersion of their hosts (e.g. Nieberding *et al*., 2004; Nieberding and Olivieri, 2007; Verneau *et al*., 2009). Moreover, an unusual recent distribution of host-specific parasites may serve as a good indicator of the human-induced introduction of invasive species into non-native regions (Benovics *et al*., 2018; Šimková *et al*., 2018; Wilson *et al*., 2019).

The Apennine Peninsula is one of the regions in Europe which has experienced numerous introductions of non-native species over the last few decades (Gherardi *et al*., 2008; Nocita *et al*., 2017; Volta *et al*., 2018). According to Gherardi *et al*. (2008), approximately 46% of freshwater fish species are non-native and their presence in this peninsula was induced by humans. Moreover, anthropogenic modifications of habitats significantly altered the natural abundance and composition of native fish fauna (Bianco, 1995). Gherardi *et al*. (2008) also hypothesized that with recent trends in introduction (either intentional to add commercially ‘more attractive’ fish into Italian rivers, or unintentional *via* ballast water exchange), the local fauna will become even more homogenized in the future. Currently, the northern Apennine Peninsula is characterized by two main ichthyogeographic districts: Padano-Venetian and Tuscano-Latium; each reflecting the recent distribution of particular endemic species (Bianco, 1990, 1995). The Padano-Venetian district covers the northern Adriatic basin and its faunal elements are shared with the north-western Balkans. The area of this district basically corresponds to the drainage of the Po River during the Last Glacial Maximum, when the sea level drastically regressed (Pielou, 1979; Bianco, 1990; Waelbroeck *et al*., 2002). During that time, freshwater river streams facilitated the mixing of faunas between two currently isolated regions (i.e. the north-western part of the Balkan, and the north-eastern part of the Apennine peninsulas) (Waelbroeck *et al*., 2002; Stefani *et al*., 2004). The Tuscano-Latium district spreads out along the Tyrrhenian slope of central Italy, between the River Arno in the north and the River Tiber in the south, and reflects the distribution of endemic *Squalius lucumonis* and encompasses almost exclusively endemic species (Bianco, 1990; Kottelat and Freyhof, 2007).

The Cyprinoidei [recently elevated Cyprinidae, and divided into the Leuciscidae and Cyprinidae families, following Stout *et al*. (2016), Schönhuth *et al*. (2018) and Tan and Armbruster (2018)] is the most diversified freshwater fish group in the Apennine Peninsula represented by 15 species, of which 12 are endemic. The distributions of three out of five species native to the Padano-Venetian district [namely, *Barbus plebejus*, *Rutilus aula* (Bonaparte, 1841) and *Squalius squalus*] extend up to the north-western Balkans (for the distribution ranges of species, see Kottelat and Freyhof, 2007). The other two species native to this district [i.e. *Chondrostoma soetta* and *Protochondrostoma genei* – the latter monotypic genus was only recently erected by Robalo *et al*. (2007)] are present only in the Apennine Peninsula. In contrast, *Telestes muticellus* represents a species with a relatively wide distribution range covering both the ichthyogeographical Padano-Venetian and Tuscano-Latium districts. Nonetheless, *T. muticellus* actually appears to represent a species complex that contains taxa with large genetic diversity (Stefani *et al*., 2004), putatively linked with the fragmentation of populations into refuges during the last glaciation cycles (Marchetto *et al*., 2010).

The distribution of the fish fauna, and especially cyprinoids, is well known in the Apennine Peninsula; however, knowledge about their parasite diversity is scarce. In light of the previous discussion, there are two major reasons to investigate the diversity of parasites in a given region: (1) parasites can represent a threat to native fauna (especially co-introduced parasite species), and (2) parasites can serve as a useful tool for investigating the evolutionary history of their hosts. Therefore, in the present study, we aimed to study the species diversity of parasitic helminths and investigate whether the diversity and distribution of the local parasite fauna of cyprinoid fish have been influenced by the introductions of non-native fish species into this region; i.e. that parasite assemblages also include alien parasite species. Moreover, we expect that native endemic cyprinoid fish will also harbour endemic parasite species (especially highly diversified and host-specific monogeneans), and by investigating the phylogenetic relationships of endemic parasites to congeners from different European regions, we will shed new light on the evolutionary history of their hosts.

## Material and methods

### Parasite sampling, fixation and identification

**Fig. 1.**
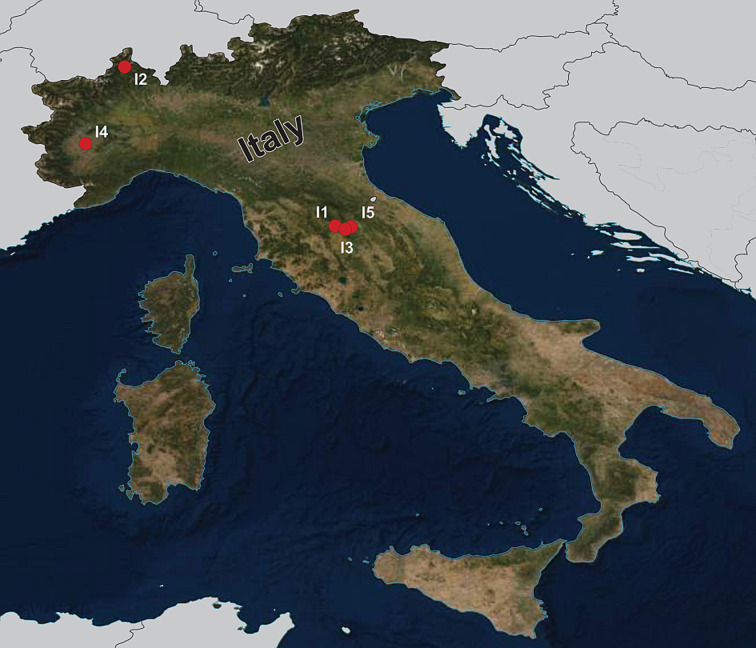
Map of the collection sites in northern Italy.

During the summers of 2015 and 2018, a total of 92 fish specimens belonging to ten species were collected from five localities in northern Italy (Fig. 1) and examined for metazoan parasites. In addition, ten specimens of *B. plebejus* and six specimens of *S. squalus* were collected by a local fisherman from the uncertain site on Po River (Table 1). Fish species were determined using Kottelat and Freyhof (2007). Prior to parasitological dissection, live fish were kept in aerated holding barrels with river water from the collection site. Fishes were paralysed and then sacrificed by severing the spinal cord. All applicable institutional, national and international guidelines for the care and use of animals were followed. The study was approved by the Animal Care and Use Committee of the Faculty of Science, Masaryk University in Brno (Czech Republic).

**Table 1.** List of investigated cyprinoid species with their respective collection sites, and list of parasite species collected from each host species divided into major taxonomical groups

Host	LocID	Locality	Coordinates	*N*	NSP	Monogenea (*Dactylogyrus*, *Gyrodactylus* and *Paradiplozoon*)	Nematoda	Digenea	Cestoda
*Alburnus arborella* (Bonaparte, 1841)	I1	canale maestro de la Chiana, Chuisa dei Capannoi, Arno basis	43°29′31.07″N 11°48′39.09″E	10	6	D. alatus*, *D. rarissimus*, *G. gobii*, *G. laevis*	*Pseudocapillaria tormentosa*, *Rhabdochona hellichi*	*–*	*–*
*Barbus caninus* Bonaparte, 1839	I2	Melezzo River, Masera	46°08′00.45″N 08°19′20.51″E	10	5	*G. barbi*, *G. katharineri*, *G. lamberti*, *Gyrodactylus cf. markewitschii*	*–*	*–*	*Bathybothrium rectangulum*
*Barbus plebejus* Bonaparte, 1839	*–*	Po, between Verono and Modena	N/A	10	6	D. achmerowi*, D. falciformis*, D. vastator*, *D. vistulae*, *G. gobii*, *G. katharineri*	*–*	*–*	*–*
*Barbus tyberinus* Bonaparte, 1839	I3	Torrente Cerfone, Intoppo	43°26′12.03″N 11°58′33.00″E	5	6	*D. crivellius*, *D. balkanicus*, *D. carpathicus*, *G. markewitschii*, *G. rugiensis*	*–*	*–*	*Bathybothrium rectangulum*
*Chondrostoma soetta* Bonaparte, 1840	I4	Carmagnola, Cave Germaire	44°51′42.96″N 07°40′26.33″E	5	3	*D. auritus*, *D. sphyrna*	*Molnaria intestinalis*	*–*	*–*
*Protochondrostoma genei* (Bonaparte, 1839)	I5	Torrente Cerfone, Le Ville	43°28′42.00″N 12°04′25.03″E	9	6	*D. conchatus*, *D. vistulae*, *D. vranoviensis*, *G. derjavini*, *G. schulmani*, *P. megan*	*–*	*–*	*–*
*Rutilus rubilio* (Bonaparte, 1837)	I3	Torrente Cerfone, Intoppo	43°26′12.03″N 11°58′33.00″E	10	7	*D. nanus*, *D. rarissimus*, *D. sphyrna*, *D. vistulae*, *G. gobii*, *G. laevis*, *P. megan*	*–*	*–*	*–*
*Squalius lucumonis* (Bianco, 1983)	I3	Torrente Cerfone, Intoppo	43°26′12.03″N 11°58′33.00″E	8	11	*D. ergensi*, *D. prostae*, *D. vistulae*, *D. vranoviensis*, *D. yinwenyingae*, *G. laevis*, *G. lamberti*, *G. lomi*, *G. vimbi*, *P. megan*	*Pseudocapillaria tormentosa*	*–*	*–*
*Squalius squalus* (Bonaparte, 1837)	*–*	Po, between Verono and Modena	N/A	6	5	*D. prostae*, *D. vistulae*, *G. laevis*, *G. lomi*, *G. vimbi*	*–*	*–*	*–*
	I3	Torrente Cerfone, Intoppo	43°26′12.03″N 11°58′33.00″E	2	6	*D. folkmanovae*, *D. prostae*, *D. vistulae*, *D. vranoviensis*, *G. gobii*, *P. megan*	*–*	*–*	*–*
*Telestes muticellus* (Bonaparte, 1837)	I3	Torrente Cerfone, Intoppo	43°26′12.03″N 11°58′33.00″E	7	7	*D. opertus*, *D. vistulae*, *G. laevis*, *G. lomi*, *G. longoacuminatus*, *G. prostae*	*–*	*Diplostomum spathaceum*	*–*
	I2	Melezzo River, Masera	46°08′00.45″N 08°19′20.51″E	10	5	*D. conchatus*, *D. sagittarius*, *G. laevis*, *G. lamberti*, *G. longoacuminatus*	*–*	*–*	*–*

LocID, codes of collection sites corresponding to the codes in Fig. 1; *N*, number of processed fish specimens; NSP, number of parasite species; dashes (*–*) indicate that no parasite species of the given taxa was found. Potentially alien species are marked by asterisks (following Galli *et al*., 2007).

Fish were dissected using the standard method described in Ergens and Lom (1970) and individual organs were observed under a stereomicroscope. Monogenean parasites were collected from fish skin, fins, gills and head cavities. Eyes, brain and muscles were checked for the presence of metacercariae of digeneans. Intestine and abdominal internal organs (i.e. gallbladder, gas bladder, liver and spleen) were checked for the presence of acanthocephalans, cestodes, digeneans and nematodes.

Monogenean parasites (*Dactylogyrus* spp., *Gyrodactylus* spp. and *Paradiplozoon* spp.) were mounted on slides (completely flattened under coverslip pressure) and fixed in a mixture of glycerine and ammonium picrate (Malmberg, 1957). For *Dactylogyrus*, at least five specimens of each species were cut in half, and one part (usually the half with the attachment organ) was preserved in 96% ethanol for further molecular analyses. The remaining body part was mounted on a slide for species determination by means of morphology. The body half used for the morphological study was later deposited (one per species) as a hologenophore [see Pleijel *et al*. (2008) for terminology] in the helminthological collection of the Institute of Parasitology of the Czech Academy of Sciences in České Budějovice (IPCAS). Species determination was performed according to the size and shape of the sclerotized elements of the attachment organ and copulatory organs following Pugachev *et al*. (2009). Endoparasitic helminths were removed from hosts and preserved in 70% ethanol. Prior to fixation in Canada balsam, cestodes and digeneans were stained in iron acetocarmine, following the protocol of Georgiev *et al*. (1986). Species determination followed Kuchta *et al*. (2008) and Gibson *et al*. (2002), respectively. Nematodes were mounted on slides and covered in a mixture of glycerine and water (in the ratio 3:7), and cleared by gradually increasing the volume of glycerol (according to Moravec, 2013). The species determination of nematode parasites followed Moravec (2013). Basic epidemiological data, i.e. prevalence, mean abundance, and minimum and maximum intensities of infection, were calculated for each parasite species (Table 2) according to Bush *et al*. (1997). Prevalence, as the percentage of fish infected by a given parasite species, and mean abundance, as the mean number of parasite specimens per individual host taking into account both infected and uninfected hosts, were calculated. Following the suggestion of Rózsa *et al*. (2000) for interpreting epidemiological data, a confidence interval at the level of 95% was calculated for mean abundance.

**Table 2.** Calculations of epidemiological data for parasite species

Species (*N*)	*Alburnus arborella* (10)			*Barbus caninus* (10)			*Barbus plebejus* (10)			*Barbus tyberinus* (5)			*Chondrostoma soetta* (5)			*Protochondrostoma genei* (9)			*Rutilus rubilio* (10)			*Squalius lucumonis* (8)			*Squalius squalus* (8)			*Telestes muticellus* (17)		
Epidemiological character	P	A	I	P	A	I	P	A	I	P	A	I	P	A	I	P	A	I	P	A	I	P	A	I	P	A	I	P	A	I
*Dactylogyrus achmerowi Gussev, 1955	*–*	*–*	*–*	*–*	*–*	*–*	10	0.10 (0.20)	1	*–*	*–*	*–*	*–*	*–*	*–*	*–*	*–*	*–*	*–*	*–*	*–*	*–*	*–*	*–*	*–*	*–*	*–*	*–*	*–*	*–*
*Dactylogyrus alatus Linstow, 1878	70	0.90 (0.46)	1*–*2	*–*	*–*	*–*	*–*	*–*	*–*	*–*	*–*	*–*	*–*	*–*	*–*	*–*	*–*	*–*	*–*	*–*	*–*	*–*	*–*	*–*	*–*	*–*	*–*	*–*	*–*	*–*
*Dactylogyrus balkanicus* Dupont and Lambert, 1986	*–*	*–*	*–*	*–*	*–*	*–*	*–*	*–*	*–*	100	22.60 (9.83)	5*–*34	*–*	*–*	*–*	*–*	*–*	*–*	*–*	*–*	*–*	*–*	*–*	*–*	*–*	*–*	*–*	*–*	*–*	*–*
*Dactylogyrus carpathicus* Zachvatkin, 1951	*–*	*–*	*–*	*–*	*–*	*–*	*–*	*–*	*–*	80	1.80 (0.96)	2*–*3	*–*	*–*	*–*	*–*	*–*	*–*	*–*	*–*	*–*	*–*	*–*	*–*	*–*	*–*	*–*	*–*	*–*	*–*
*Dactylogyrus conchatus n. sp.*	*–*	*–*	*–*	*–*	*–*	*–*	*–*	*–*	*–*	*–*	*–*	*–*	*–*	*–*	*–*	56	1.44 (1.03)	1–4	–	–	–	–	–	–	–	–	–	41	1.24 (0.88)	1–3
*Dactylogyrus crivellius* Dupont and Lambert, 1986	*–*	*–*	*–*	*–*	*–*	*–*	*–*	*–*	*–*	100	46.40 (17.93)	15*–*67	*–*	*–*	*–*	*–*	*–*	*–*	*–*	*–*	*–*	*–*	*–*	*–*	*–*	*–*	*–*	*–*	*–*	*–*
*Dactylogyrus ergensi* Molnár, 1964	*–*	*–*	*–*	*–*	*–*	*–*	*–*	*–*	*–*	*–*	*–*	*–*	*–*	*–*	*–*	*–*	*–*	*–*	*–*	*–*	*–*	37.5	1.38 (1.61)	1*–*6	*–*	*–*	*–*	*–*	*–*	*–*
*Dactylogyrus falciformis Akhmerov, 1952	*–*	*–*	*–*	*–*	*–*	*–*	10	0.20 (0.39)	2	*–*	*–*	*–*	*–*	*–*	*–*	*–*	*–*	*–*	*–*	*–*	*–*	*–*	*–*	*–*	*–*	*–*	*–*	*–*	*–*	*–*
*Dactylogyrus folkmanovae* Ergens, 1956	*–*	*–*	*–*	*–*	*–*	*–*	*–*	*–*	*–*	*–*	*–*	*–*	*–*	*–*	*–*	*–*	*–*	*–*	*–*	*–*	*–*	*–*	*–*	*–*	12.5	0.75 (1.47)	6	*–*	*–*	*–*
*Dactylogyrus globulatus n. sp.*	*–*	*–*	*–*	*–*	*–*	*–*	*–*	*–*	*–*	*–*	*–*	*–*	60	11.60 (11.57)	11*–*32	*–*	*–*	*–*	*–*	*–*	*–*	*–*	*–*	*–*	*–*	*–*	*–*	*–*	*–*	*–*
*Dactylogyrus nanus* Dogiel and Bychowsky, 1934	*–*	*–*	*–*	*–*	*–*	*–*	*–*	*–*	*–*	*–*	*–*	*–*	*–*	*–*	*–*	*–*	*–*	*–*	100	2.70 (1.31)	1*–*8	*–*	*–*	*–*	*–*	*–*	*–*	*–*	*–*	*–*
*Dactylogyrus opertus n. sp.*	*–*	*–*	*–*	*–*	*–*	*–*	*–*	*–*	*–*	*–*	*–*	*–*	*–*	*–*	*–*	*–*	*–*	*–*	*–*	*–*	*–*	*–*	*–*	*–*	*–*	*–*	*–*	29.4	0.41 (0.34)	1*–*2
*Dactylogyrus prostae* Molnár, 1964	*–*	*–*	*–*	*–*	*–*	*–*	*–*	*–*	*–*	*–*	*–*	*–*	*–*	*–*	*–*	*–*	*–*	*–*	*–*	*–*	*–*	25	0.50 (0.64)	2	25	2.38 (4.12)	2*–*17	*–*	*–*	*–*
*Dactylogyrus rarissimus* Gussev, 1966	30	0.30 (0.30)	1	*–*	*–*	*–*	*–*	*–*	*–*	*–*	*–*	*–*	*–*	*–*	*–*	*–*	*–*	*–*	60	0.80 (0.57)	1*–*3	*–*	*–*	*–*	*–*	*–*	*–*	*–*	*–*	*–*
*Dactylogyrus sagittarius n. sp.*	*–*	*–*	*–*	*–*	*–*	*–*	*–*	*–*	*–*	*–*	*–*	*–*	*–*	*–*	*–*	*–*	*–*	*–*	*–*	*–*	*–*	*–*	*–*	*–*	*–*	*–*	*–*	23.5	0.53 (0.53)	1*–*4
*Dactylogyrus sphyrna* Linstow, 1878	*–*	*–*	*–*	*–*	*–*	*–*	*–*	*–*	*–*	*–*	*–*	*–*	80	10.60 (8.89)	6*–*27	*–*	*–*	*–*	60	1.50 (1.53)	1*–*8	*–*	*–*	*–*	*–*	*–*	*–*	*–*	*–*	*–*
*Dactylogyrus vastator Nybelin, 1924	*–*	*–*	*–*	*–*	*–*	*–*	20	0.20 (0.26)	1	*–*	*–*	*–*	*–*	*–*	*–*	*–*	*–*	*–*	*–*	*–*	*–*	*–*	*–*	*–*	*–*	*–*	*–*	*–*	*–*	*–*
*Dactylogyrus vistulae* Prost, 1957	*–*	*–*	*–*	*–*	*–*	*–*	20	0.20 (0.26)	1	*–*	*–*	*–*	*–*	*–*	*–*	44.4	0.56 (0.47)	1*–*2	30	0.30 (0.30)	1	62.5	2.13 (1.90)	1*–*8	87.5	4.88 (2.86)	2*–* 12	53.8	2.65 (2.06)	1*–*13
*Dactylogyrus vranoviensis* Ergens, 1956	*–*	*–*	*–*	*–*	*–*	*–*	*–*	*–*	*–*	*–*	*–*	*–*	*–*	*–*	*–*	33.3	0.67 (0.86)	1*–*4	*–*	*–*	*–*	50	0.63 (0.73)	1*–*3	12.5	0.13 (0.24)	1	*–*	*–*	*–*
*Dactylogyrus yinwenyingae* Gussev, 1962	*–*	*–*	*–*	*–*	*–*	*–*	*–*	*–*	*–*	*–*	*–*	*–*	*–*	*–*	*–*	*–*	*–*	*–*	*–*	*–*	*–*	25	0.25 (0.32)	1	*–*	*–*	*–*	*–*	*–*	*–*
*Gyrodactylus barbi* Ergens, 1976	*–*	*–*	*–*	90	4.20 (1.59)	2*–*9	*–*	*–*	*–*	*–*	*–*	*–*	*–*	*–*	*–*	*–*	*–*	*–*	*–*	*–*	*–*	*–*	*–*	*–*	*–*	*–*	*–*	*–*	*–*	*–*
*Gyrodactylus derjavini* Mikailov, 1975	*–*	*–*	*–*	*–*	*–*	*–*	*–*	*–*	*–*	*–*	*–*	*–*	*–*	*–*	*–*	33.3	0.67 (0.65)	2	*–*	*–*	*–*	*–*	*–*	*–*	*–*	*–*	*–*	*–*	*–*	*–*
*Gyrodactylus gobii* Schulman, 1953	60	3.40 (2.07)	3*–*9	*–*	*–*	*–*	40	0.60 (0.52)	1*–*2	*–*	*–*	*–*	*–*	*–*	*–*	*–*	*–*	*–*	50	1.1 (0.90)	1*–*4	*–*	*–*	*–*	12.5	0.13 (0.24)	1	*–*	*–*	*–*
*Gyrodactylus katharineri* Malmberg, 1964	*–*	*–*	*–*	90	7.30 (3.47)	1*–*16	30	0.30 (0.30)	1	*–*	*–*	*–*	*–*	*–*	*–*	*–*	*–*	*–*	*–*	*–*	*–*	*–*	*–*	*–*	*–*	*–*	*–*	*–*	*–*	*–*
*Gyrodactylus laevis* Malmberg, 1957	60	1.30 (0.88)	1*–*4	*–*	*–*	*–*	*–*	*–*	*–*	*–*	*–*	*–*	*–*	*–*	*–*	*–*	*–*	*–*	20	0.20 (0.26)	1	*–*	*–*	*–*	12.5	0.13 (0.24)	1	23.5	0.35 (0.33)	1*–*2
*Gyrodactylus lamberti* Ergens, 1990	*–*	*–*	*–*	30	0.70 (0.97)	1*–*5	*–*	*–*	*–*	*–*	*–*	*–*	*–*	*–*	*–*	*–*	*–*	*–*	*–*	*–*	*–*	12.5	0.13 (0.24)	1	*–*	*–*	*–*	52.94	3.00 (1.68)	2*–*11
*Gyrodactylus lomi* Ergens and Gelnar, 1988	*–*	*–*	*–*	*–*	*–*	*–*	*–*	*–*	*–*	*–*	*–*	*–*	*–*	*–*	*–*	*–*	*–*	*–*	*–*	*–*	*–*	37.5	3.38 (3.78)	3*–*13	62.5	0.63 (0.36)	1	17.64	0.41 (0.58)	1*–*5
*Gyrodactylus longoacuminatus* Zitnan, 1964	*–*	*–*	*–*	*–*	*–*	*–*	*–*	*–*	*–*	*–*	*–*	*–*	*–*	*–*	*–*	*–*	*–*	*–*	*–*	*–*	*–*	*–*	*–*	*–*	*–*	*–*	*–*	70.58	9.53 (4.31)	1*–*27
*Gyrodactylus markewitschii* Kulakowskaja, 1951	*–*	*–*	*–*	*–*	*–*	*–*	*–*	*–*	*–*	40	1.20 (1.90)	1*–*5	*–*	*–*	*–*	*–*	*–*	*–*	*–*	*–*	*–*	*–*	*–*	*–*	*–*	*–*	*–*	*–*	*–*	*–*
*Gyrodactylus prostae* Ergens, 1963	*–*	*–*	*–*	*–*	*–*	*–*	*–*	*–*	*–*	*–*	*–*	*–*	*–*	*–*	*–*	*–*	*–*	*–*	*–*	*–*	*–*	*–*	*–*	*–*	*–*	*–*	*–*	5.88	0.06 (0.12)	1
*Gyrodactylus rugiensis* Glaser, 1974	*–*	*–*	*–*	*–*	*–*	*–*	*–*	*–*	*–*	20	0.20 (0.39)	1	*–*	*–*	*–*	*–*	*–*	*–*	*–*	*–*	*–*	*–*	*–*	*–*	*–*	*–*	*–*	*–*	*–*	*–*
*Gyrodactylus schulmani* Ling, 1962	*–*	*–*	*–*	*–*	*–*	*–*	*–*	*–*	*–*	*–*	*–*	*–*	*–*	*–*	*–*	11.1	0.11 (0.22)	1	*–*	*–*	*–*	*–*	*–*	*–*	*–*	*–*	*–*	*–*	*–*	*–*
*Gyrodactylus vimbi* Schulman, 1953	*–*	*–*	*–*	*–*	*–*	*–*	*–*	*–*	*–*	*–*	*–*	*–*	*–*	*–*	*–*	*–*	*–*	*–*	*–*	*–*	*–*	12.5	0.25 (0.49)	2	12.5	0.13 (0.24)	1	*–*	*–*	*–*
*Gyrodactylus cf. markewitschii*	*–*	*–*	*–*	40	4.20 (5.00)	1*–*24	*–*	*–*	*–*	*–*	*–*	*–*	*–*	*–*	*–*	*–*	*–*	*–*	*–*	*–*	*–*	*–*	*–*	*–*	*–*	*–*	*–*	*–*	*–*	*–*
*Paradiplozoon megan* (Bychowsky and Nagibina, 1959)	*–*	*–*	*–*	*–*	*–*	*–*	*–*	*–*	*–*	*–*	*–*	*–*	*–*	*–*	*–*	33.3	1.78 (2.14)	1*–*8	10	0.20 (0.39)	2	62.5	1.50 (1.43)	1*–*6	12.5	0.88 (1.71)	7	*–*	*–*	*–*
*Molnaria intestinalis* (Dogiel and Bychowsky, 1934)	*–*	*–*	*–*	*–*	*–*	*–*	*–*	*–*	*–*	*–*	*–*	*–*	20	2.60 (5.10)	13	*–*	*–*	*–*	*–*	*–*	*–*	*–*	*–*	*–*	*–*	*–*	*–*	*–*	*–*	*–*
*Pseudocapillaria tormentosa* (Durjadin, 1843)	10	0.20 (0.39)	2	*–*	*–*	*–*	*–*	*–*	*–*	*–*	*–*	*–*	*–*	*–*	*–*	*–*	*–*	*–*	*–*	*–*	*–*	12.5	0.25 (0.49)	2	*–*	*–*	*–*	*–*	*–*	*–*
*Rhabdochona helichii* (Srámek, 1901)	30	0.60 (0.60)	2	*–*	*–*	*–*	*–*	*–*	*–*	*–*	*–*	*–*	*–*	*–*	*–*	*–*	*–*	*–*	*–*	*–*	*–*	*–*	*–*	*–*	*–*	*–*	*–*	*–*	*–*	*–*
*Diplostomum spathaceum* (Rudolphi, 1819)	*–*	*–*	*–*	*–*	*–*	*–*	*–*	*–*	*–*	*–*	*–*	*–*	*–*	*–*	*–*	*–*	*–*	*–*	*–*	*–*	*–*	*–*	*–*	*–*	*–*	*–*	*–*	17.64	1.88 (2.14)	6*–*15
*Bathybothrium rectangulum* (Bloch, 1782)	*–*	*–*	*–*	50	2.80 (3.09)	1*–*14	*–*	*–*	*–*	20	0.20 (0.39)	1	*–*	*–*	*–*	*–*	*–*	*–*	*–*	*–*	*–*	*–*	*–*	*–*	*–*	*–*	*–*	*–*	*–*	*–*

P, prevalence; A, mean abundance (confidence interval for level 95%); I, range of intensity of infection (min–max). Potentially alien species are marked by asterisks (following Galli *et al*., 2007).

### DNA extraction, amplification and sequencing

The extraction of genomic DNA from *Dactylogyrus* parasites was performed using a commercially produced extraction kit (DNeasy Blood & Tissue Kit, Qiagen, Hilden, Germany) following the manufacturer's protocol. Two genomic DNA regions were amplified, the first one covering the partial gene coding for 18S rRNA, the complete ITS1 region and the partial gene coding for 5.8S rRNA (hereinafter abbreviated as 18S, ITS1 and 5.8S), and the second one covering the partial gene coding for 28S rRNA (hereinafter abbreviated as 28S). The first region was amplified using the primers S1 (forward, 5′-ATTCCGATAACGAACGAGACT-3′) and IR8 (reverse, 5′-GCTAGCTGCGTTCTTCATCGA-3′), which anneal to the segments of DNA coding for 18S and 5.8S rRNA, respectively (Šimková *et al*., 2003). The amplification reaction followed the protocol optimized by Benovics *et al*. (2018). The latter region was amplified using the primers C1 (forward, 5′-ACCCGCTGAATTTAAGCA-3′) and D2 (reverse, 5′-TGGTCCGTGTTTCAAGAC-3′) (Hassouna *et al*., 1984), following the protocol of Benovics *et al*. (2020). The PCR products (~1000 for 18S, ITS1 and 5.8S, and ~800 bp for partial 28S) were checked on 1% agarose gel and purified using the ExoSAP-IT kit (Ecoli, Bratislava, Slovakia) following the standard protocol. The purified products were directly sequenced using the same primers as for PCR and BigDye Terminator Cycle Sequencing kit (Applied Biosystems, Prague, Czech Republic). Sequencing was performed on an ABI 3130 Genetic Analyzer (Applied Biosystems).

### Morphometric data and species description

**Fig. 2.**
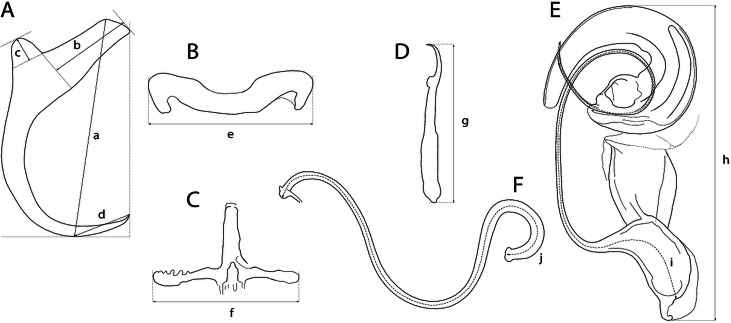
Scheme of measurements for sclerotized structures of *Dactylogyrus* spp. A, anchor (a – total length, b – inner root length, c – outer root length, d – point length); B, dorsal bar (e – width); C, ventral bar (f – width); D, hook (g – length); E, male copulatory organ (h – total straight length, i – tube curved length); F, vagina (j – curved length).

The mounted monogeneans (or their parts) were studied using an Olympus BX 61 microscope equipped with phase-contrast optics. Drawings were made with the aid of a drawing attachment and edited with a graphic tablet compatible with Adobe Illustrator and Adobe Photoshop. Measurements were taken using digital image analysis (Stream Motion, version 1.9.2) and are given in micrometres [mean followed by the range and number of specimens measured (*n*) in parentheses]. The measurement scheme for the sclerotized structures of *Dactylogyrus* is shown in Fig. 2. The numbering of hook pairs (in Roman numerals) follows that recommended by Mizelle (1936). The male copulatory organ is abbreviated as MCO.

The parasitological material was deposited in the Helminthological collection of the Institute of Parasitology, Academy of Sciences of the Czech Republic, České Budějovice, Czech Republic (IPCAS). The material was remounted in Canada balsam (according to Ergens, 1969) prior to depositing. To comply with the regulations set out in article 8.5 of the amended 2012 version of the International Code of Zoological Nomenclature (ICZN, 2012), details of the new species have been submitted to ZooBank. Note that the authorities of the new taxa described below are Francová & Benovics (according to the ICZN, 2012).

### Phylogenetic analyses

To infer the phylogenetic positions of new *Dactylogyrus* species, three genetic markers were selected for phylogenetic analyses; partial genes coding for 18S rRNA and 28S rRNA, and the ITS1 region. In addition to the newly obtained sequences, sequences corresponding to already described *Dactylogyrus* species were retrieved from GenBank (see accession numbers in the Supplementary data, Table S1). The *Dactylogyrus* species for phylogenetic analyses were selected in order to investigate the phylogenetic relationships between species from two southern European peninsulas with a historical connection (i.e. the Apennine and Balkan peninsulas), and their relationships to *Dactylogyrus* species parasitizing cyprinoids with a wide distribution range in Europe, especially in the geographically proximal central Europe. For *Dactylogyrus* species found on cyprinoids of the Apennine Peninsula and previously reported on cyprinoid species in other European regions (the Balkans or Central Europe, Šimková *et al*., 2004; Benovics *et al*., 2017, 2018; Řehulková *et al*., 2020), already published DNA sequences were retrieved from GenBank. DNA sequences were concatenated and aligned using the fast Fourier transform algorithm implemented in MAFFT (Katoh *et al*., 2002) and subsequently manually trimmed to unify the lengths of all used sequences. The final alignment was built from 42 *Dactylogyrus* species (38 previously described, and 4 new) of various cyprinoid taxa from the Apennine and Balkan peninsulas, and central Europe. Following the phylogenetic reconstruction of Benovics *et al*. (2018), three common *Dactylogyrus* species of *Carassius gibelio* (Bloch, 1782) (namely *D. anchoratus*, *D. formosus* and *D. vastator*) were used as the outgroup for rooting the phylogenetic trees. The aligned dataset was treated as partitioned, each partition associated with an individual genetic marker, i.e. 18S, 28S, ITS1. General time-reversible model was applied to each partition. Phylogenetic analyses were computed by means of maximum likelihood (ML) and Bayesian inference (BI) methods, using RaxML v 8.2.11 (Stamatakis, 2006, 2014) and MrBayes v 3.2.6 (Ronquist *et al*., 2012), respectively. Internal node support for the ML tree was assessed by running 1000 bootstrap pseudoreplicates. Two parallel runs, each with four Markov chains, were executed for the BI analysis and run for 10^7^ generations. Trees were sampled every 10^2^ generations and the first 30% of the resulting trees were discarded as initial burn-in after checking convergence of runs in Tracer v 1.7.1 (Rambaut *et al*., 2018). Posterior probabilities were calculated as the frequencies of samples recovering particular clades.

## Results

### Diversity of helminth parasites

A total of 41 helminth species were collected and identified (Table 2), with *D. vistulae* as the most prevalent parasite species, and *G. lomi* as the most abundant species. Parasites communities with the greatest species richness were harboured by *S. lucumonis* from the Torrente Cerfone locality (11 helminth species). In contrast, only three helminth species were collected from *C. soetta* (Carmagnola). The basic epidemiological parameters, i.e. prevalence, mean abundance with a confidence interval at the level of 0.95, and minimum and maximum intensity of infection, are presented for each parasite species in Table 2. In general, monogeneans exhibited the highest species diversity. Thirty-one known monogenean species and four newly-described species were found on the gills and in the nasal cavity of fish. Concerning *Dactylogyrus*, out of 20 species, *D. vistulae* was reported on the widest range of host species (collected from six host species) exhibiting the highest prevalence and highest mean abundance on *S. squalus.* The second most abundant monogenean genus, *Gyrodactylus*, was represented by 14 species, of which *G. gobii* and *G. laevis* were collected from the highest number of host species (four species). Only one diplozoid species (*Paradiplozoon megan*) was found, parasitizing all four species collected from Torrente Cerfone, with the highest prevalence on *S. lucumonis*. However, *P. megan* achieved the highest mean abundance and intensity of infection on *P. genei*. Only one species of eye fluke (metacercariae of *Diplostomum spathaceum*) was found on two specimens of *T. muticellus* from Torrente Cerfone with moderate abundance. No other digeneans were detected during this survey. Intestinal helminths were represented by three species of nematodes (*Molnaria intestinalis*, *Pseudocapillaria tormentosa* and *Rhabdochona hellichi*) and a single cestode species belonging to the monotypic genus *Bathybothrium* (*Bathybothrium rectangulum*) collected from two *Barbus* species (*B. caninus* and *B. tyberinus*). However, only a single specimen of *B. rectangulum* was collected from *B. tyberinus*.

### Morphological and molecular characterization of the new species

The new *Dactylogyrus* species described in this study were classified into two morphological groups according to Pugachev *et al*. (2009) on the basis of the morphology of the sclerotized parts of the reproductive organs – the MCO and the vagina.

Three of the new *Dactylogyrus* species (i.e. *D. globulatus* n. sp., *D. conchatus* n. sp. and *D. sagittarius* n. sp.) belonged to the ‘ergensi’ (or sometimes termed ‘chondrostomi’) morphological group, i.e. a group of *Dactylogyrus* species having an MCO with a complex accessory piece, comprising ‘a broad tongue-shaped lobe directed backwards along the circle of the curved tube’, and a vagina in the form of an elongate tube. Of the ‘ergensi’ group species, the three new species most resemble those possessing a three-armed or cross-shaped ventral bar (with a simple, not bifurcated, anterior arm): *Dactylogyrus elegantis* Gussev, 1966; *D. dirigerus* Gussev, 1966; *D. ergensi* Molnár, 1964; *D. nybelini* Markewitsch, 1933; *D. naviculoides* Ergens, 1956; *D. soufii* Lambert, 1977; *D. caucasicus* Mikailov et Shaova, 1973; and *D. dimitrovae* Kakacheva-Avramova, 1972.

*Dactylogyrus ergensi*, collected from *Chondrostoma nasus* in the Danube River in Hungary, was described by Molnár (1964). Since then, specimens from several distant localities displaying the same basic morphology but also high morphometrical variability in their sclerotized structures have been assigned to this species (Pugachev *et al*., 2009); we consider these specimens likely representing the members of more than one species. The morphology of the sclerotized structures of the present new species was compared with that of *D. ergensi* using an original description of the latter species (Molnár, 1964).

The last of the four *Dactylogyrus* species described in this study (i.e. *D. opertus* n. sp.) was assigned to the ‘nanus’ morphological group, specifically to the subgroup of species displaying the following features: an MCO comprising a simple copulatory tube, smoothly curved all along its length from the initial part (or more curved distally), and terminally enclosed with a markedly enlarged distal part (a sheath) of the accessory piece; a vagina present as a short or medium-size bent cylindrical tube, closed with a cap (i.e. the subgroup including *Dactylogyrus nanus* Dogiel et Bychowsky, 1934; *D. nanoides* Gussev, 1966; and *D. suecicus* Nybelin, 1937).

Descriptions of all new *Dactylogyrus* species are given below.

**Dactylogyridea Bychowsky, 1937**

**Dactylogyridae Bychowsky, 1933**

***Dactylogyrus* Diesing, 1850**

**Fig. 3.**
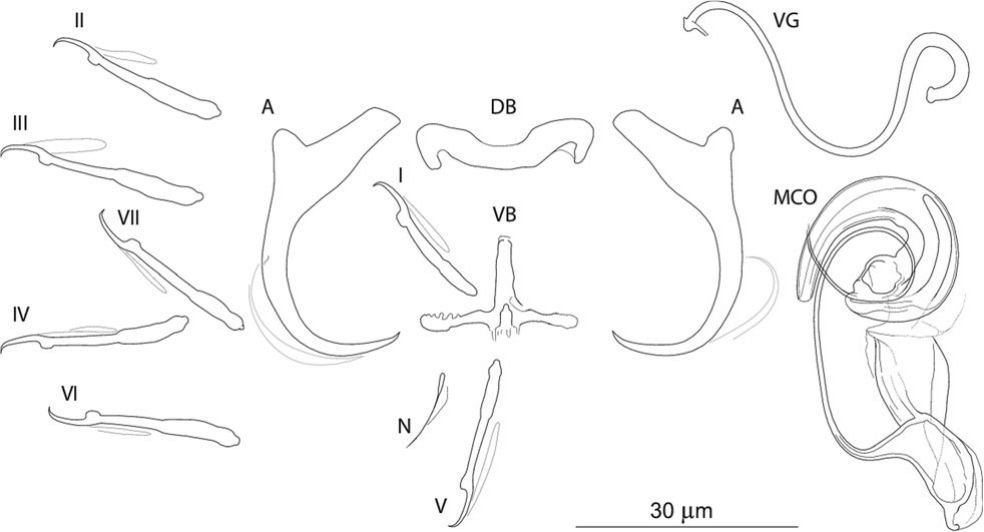
Sclerotized structures of *Dactylogyrus globulatus n. sp. ex Chondrostoma soetta*. A, anchor; DB, dorsal bar; VB, ventral bar; N, needle; I–VII, hooks; VG, vagina; MCO, male copulatory organ.

***Dactylogyrus globulatus* n. sp. (**Fig. 3**)**

urn:lsid:zoobank.org:act:CAECC9DF-17D4-4699-9A52-2BF8C4373B30

**Type host and locality:** *Chondrostoma soetta* Bonaparte, 1840; Carmagnola, Cave Germaire (44°51′42.96″N, 07°40′26.33″E).

**Site on host:** Gill lamellae.

**Type and voucher material:** IPCAS M-753 (holotype; five paratypes; one hologenophore voucher).

**Representative DNA sequences:** A nucleotide sequence of partial gene for 28S rRNA (789 bp long; MW443032), and nucleotide sequences representing a fragment (961 bp long; MW443035) including partial gene for 18S rRNA (487 bp), and the ITS1 region (474 bp). No intraspecific variability was found (seven specimens were analysed).

**Etymology:** The specific name is derived from Latin (*globus* = ball, sphere, globe; treated as an adjective) and refers to the shape of the distal part of the MCO accessory piece which seems to be rolled into a ball.

**Description** (based on eight specimens): Body length 469 (437–531; *n* = 4); greatest width 70 (59–86; *n* = 4). Haptor with one pair of anchors (dorsal). Anchors with moderately developed roots (inner root longer, more or less flattened terminally); roots forming an angle of/>90°; shaft evenly curved to point; point with slightly recurved tip; total length 32 (30–34; *n* = 8); inner root length 12 (11–13; *n* = 8); outer root length 4 (3–4; *n* = 8); point length 9 (8–10; *n* = 8). Anchor filaments well developed. Dorsal bar 23 (20–25; *n* = 8) wide, saddle-shaped. Ventral bar 21 (18–23; *n* = 8) wide, cross-shaped, with posterior arm markedly reduced to fringe; medial aperture open at posterior edge. One pair of needles located near the hooks of pair V. Hooks seven pairs; each with delicate point, depressed truncate thumb, shank comprised of two subunits; proximal subunit expanded, with small terminal nipple (except for hooks of pair I); hook lengths: pair I 20 (19–21; *n* = 8); pairs II–VII 25 (22–29; *n* = 8). MCO composed of basally articulated copulatory tube and accessory piece; total length 49 (48–51; *n* = 7). Copulatory tube a loose coil of one terminal ring, 93 (91–95; *n* = 6) long; base an elongate oval; shaft following S-shaped path proximally, narrowing to delicate termination. Accessory piece proximally forming membranous skirt around the margin of copulatory tube base; medial portion a folded membrane, cone-shaped, serving as a guide for distal portion; distal portion a complex tongue-shaped structure curved (backwards) along the terminal coil of the copulatory tube. Vagina-like arrow with small arrowhead and long wavy arrow shaft, 71 (67–74; *n* = 8) long.

**Remarks:** *Dactylogyrus globulatus* n. sp. is similar to *Dactylogyrus* species belonging to the ‘ergensi’ morphological group (Pugachev *et al*., 2009) (see above), including *D. conchatus* n. sp. and *D. sagittarius* n. sp. The new species most closely resembles *D. ergensi* and *D. conchatus* n. sp. – the species possessing a basically cross-shaped ventral bar. While the posterior arm ventral bar is reduced in the new species and *D. conchatus* n. sp., it is apparently more developed in *D. ergensi* [according to the drawing of Molnár (1964)], separating the new species, and also *D. conchatus* n. sp., from all other species of the ‘ergensi’ group.

On the basis of the comparison with *D. ergensi*, originally described (and depicted) by Molnár (1964), the present new species can be distinguished from the latter by the following characters: (i) dorsal anchor with shaft evenly curved to point, and slightly recurved point tip (*vs* well-differentiated and relatively long point in *D. ergensi*), (ii) dorsal and ventral haptoral bars of similar width (*vs* dorsal bar noticeably wider than ventral bar in *D. ergensi*), (iii) cross-shaped ventral bar with markedly reduced posterior arm and medial aperture open on posterior edge (*vs* cross-shaped ventral bar with elongate aperture pervading both anterior arm and relatively well-developed posterior arm in *D. ergensi*), (iv) markedly longer sclerotized part of the vagina (67–74 *vs* 24–28 *μ*m in *D. ergensi*).

The differentiation of *D. globulatus* n. sp. from *D. conchatus* n. sp. and *D. sagittarius* n. sp. is provided in the differential diagnosis for the latter two new species.

**Fig. 4.**
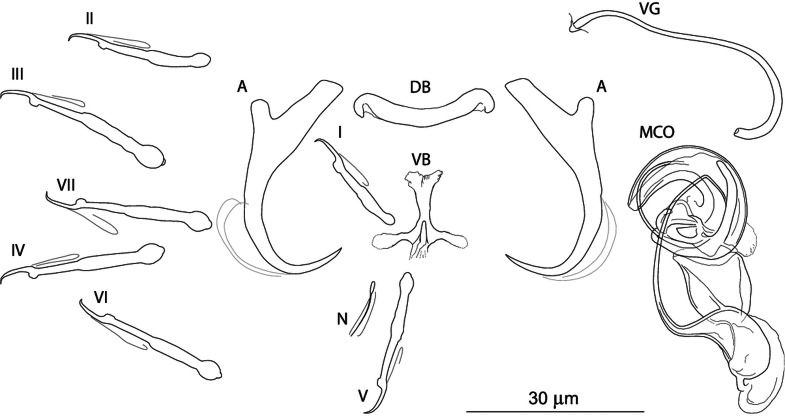
Sclerotized structures of *Dactylogyrus conchatus n. sp. ex Telestes muticellus.* A, anchor; DB, dorsal bar; VB, ventral bar; N, needle; I–VII, hooks; VG, vagina; MCO, male copulatory organ.

***Dactylogyrus conchatus* n. sp. (**Fig. 4**)**

urn:lsid:zoobank.org:act:271E1FD9-A859-427C-AC34-771B063FE05F

**Type host and locality:** *Telestes muticellus* (Bonaparte, 1837); Melezzo River, Masera (46°08′00.45″N, 08°19′20.51″E).

**Other host and locality:** *Protochondrostoma genei* (Bonaparte, 1839); Torrente Cerfone, Le Ville (43°28′42.00″N, 12°04′25.03″E).

**Site on host:** Gill lamellae.

**Type and voucher material:** IPCAS M-754 (holotype; one paratype; three vouchers; one hologenophore voucher).

**Representative DNA sequences:** A nucleotide sequence of partial gene for 28S rRNA (788 bp long; MW443033) and nucleotide sequences representing a fragment (986 bp long; MW443036) including partial gene for 18S rRNA (487 bp), the ITS1 region (493 bp) and 5.8S (6 bp). No intraspecific variability was found (nine specimens were analysed).

**Etymology:** The specific epithet is derived from Latin (*concha* = mollusc shell; treated as an adjective) and reflects the snail shell appearance of the distal potion of the MCO accessory piece.

**Description** (based on four specimens): Body length 542 (470–583; *n* = 3); greatest width 92 (78–106; *n* = 3). Haptor with one pair of anchors (dorsal). Anchors with moderately developed roots (inner root longer, terminally flattened); roots forming acute angle; medially slightly constricted bent shaft; point; total length 33 (32–33; *n* = 4); inner root length 12 (11–13; *n* = 4); outer root length 4 (4–5; *n* = 4); point length 9 (8–9; *n* = 4). Anchor filaments well developed. Dorsal bar 23 (21–24; *n* = 4) wide, saddle-shaped. Ventral bar 17 (16–17; *n* = 4) wide, cross-shaped, with anterior arm widened terminally; posterior arm markedly reduced; medial aperture open at posterior edge. One pair of needles located near hooks of pair V. Hooks seven pairs; each with delicate point, depressed truncate thumb, shank comprised of two subunits; proximal subunit expanded; terminal part of proximal subunit sometimes ball-like, with small nipple (except for hook of pair I); hook lengths: pair I 20 (19–20; *n* = 4); pairs II–VII 26 (22–31; *n* = 4). MCO comprising copulatory tube and basally articulated accessory piece; total length 47 (45–49; *n* = 5). Copulatory tube a loose coil of one terminal ring, 92 (90–93; *n* = 2) long; base an elongate oval; shaft following S-shaped path proximally, narrowing to delicate termination. Accessory piece proximally forming membranous skirt around the margin of copulatory tube base; medial portion a folded membrane, cone-shaped, serving as a guide for distal portion; distal portion a complex tongue-shaped structure curved (backwards) along the terminal coil of the copulatory tube. Vagina-like arrow with small arrowhead and long wavy arrow shaft, 63 (54–74; *n* = 5) long.

**Remarks:** *Dactylogyrus conchatus* n. sp. fits the ‘ergensi’ morphological group (Pugachev *et al*., 2009) (see above), including *D. globulatus* n. sp. and *D. sagittarius* n. sp. The new species most closely resembles *D. ergensi* and *D. globulatus* n. sp. – the species possessing a basically cross-shaped ventral bar. While the posterior arm ventral bar is reduced in the new species and *D. globulatus* n. sp., it is apparently more developed in *D. ergensi* [according to the drawing of Molnár (1964)], separating the new species, and also *D. globulatus* n. sp., from all other species of the ‘ergensi’ group.

According to the original description (and depiction) of *D. ergensi* by Molnár (1964), *D. conchatus* n. sp. can be distinguished from the latter species by the following characters: (i) cross-shaped ventral bar with markedly reduced posterior arm and with medial aperture open on posterior edge (*vs* cross-shaped ventral bar with elongate aperture pervading both anterior arm and relatively well-developed posterior arm in *D. ergensi*), and (ii) markedly longer sclerotized part of the vagina (54–74 *vs* 24–28 *μ*m in *D. ergensi*).

*Dactylogyrus conchatus* n. sp. can be separated morphologically from *D. globulatus* n. sp. on the basis of the following characters: (i) dorsal anchor possesses well-differentiated point (*vs* less differentiated point, with a slightly recurved tip in *D. globulatus* n. sp.), (ii) dorsal bar is more slender, (iii) anterior arm of ventral bar is widened terminally. Moreover, the *D. conchatus* n. sp. is also divergent from other congeners at the molecular level.

Differences between *D. conchatus* n. sp. and *D. sagittarius* n. sp. are specified in the differential diagnosis for the latter species.

**Fig. 5.**
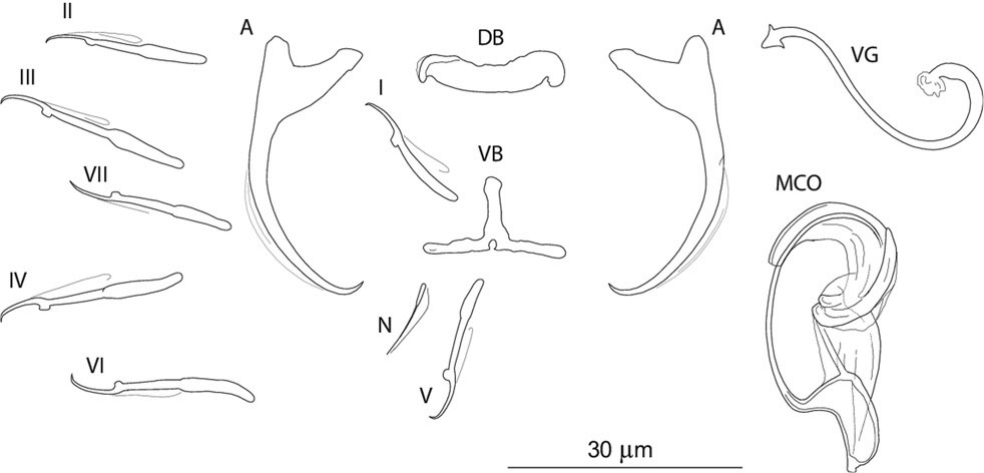
Sclerotized structures of *Dactylogyrus sagittarius n. sp. ex Telestes muticellus.* A, anchor; DB, dorsal bar; VB, ventral bar; N, needle; I–VII, hooks; VG, vagina; MCO, male copulatory organ.

***Dactylogyrus sagittarius* n. sp. (**Fig. 5**)**

urn:lsid:zoobank.org:act:3EB571AF-C1B5-4360-9E7E-CE268C7466B6

**Type host and locality:** *Telestes muticellus* (Bonaparte, 1837); Melezzo River, Masera (46°08′00.45″N, 08°19′20.51″E).

**Site on host:** Gill lamellae.

**Type and voucher material:** IPCAS M-755 (holotype; one paratype; one hologenophore voucher).

**Representative DNA sequences:** A nucleotide sequence of partial gene for 28S rRNA (789 bp long; MW443034) and nucleotide sequences representing a fragment (966 bp long; MW443037) including partial gene for 18S rRNA (487 bp), and the ITS1 region (479 bp). No intraspecific variability was found (six specimens were analysed).

**Etymology:** The specific name, a noun, is from Latin (*Sagittarius* = the archer armed with arrow), referring to the new species having an arrow-shaped vagina.

**Description** (based on five specimens): Body length 512 (485–539; *n* = 2); greatest width 59 (54–64; *n* = 2). Haptor with one pair of anchors (dorsal). Anchors with moderately developed roots (inner root slightly constricted subterminally); roots (sometimes) forming obtuse angle; elongate curved shaft; short point; total length 34 (33–35; *n* = 5); inner root length 9 (8–9; *n* = 5); outer root length 4 (3–4; *n* = 5); point length 3 (3–4; *n* = 5). Anchor filaments well developed. Dorsal bar 20 (19–22; *n* = 5) wide, saddle-shaped. Ventral bar 19 (18–20; *n* = 4) wide, three-armed, with small medial aperture open on posterior edge. One pair of needles located near hooks of pair V. Hooks seven pairs; each with delicate point, depressed truncate thumb, shank comprised of two subunits; proximal subunit expanded (except for hook of pair I); hook length (pairs I–VII) 21 (15–25; *n* = 5). MCO composed of basally articulated copulatory tube and accessory piece; total length 37 (35–41; *n*  = 3). Copulatory tube a loose coil of one terminal ring, 71 (70–72; *n* = 2) long; base an elongate oval; shaft following S-shaped path proximally, narrowing to delicate termination. Accessory piece basically comprising two portions; proximal portion membranous, folded, serving as a guide for distal portion; distal portion a complex tongue-shaped structure curved (backwards) along the terminal coil of the copulatory tube. Vagina-like arrow with small arrowhead and long wavy arrow shaft; blossom-like initial part (opening), 49 (42–55; *n* = 5) long.

**Remarks:** *Dactylogyrus sagittarius* n. sp. resembles *Dactylogyrus* species belonging to the ‘ergensi’ morphological group (Pugachev *et al*., 2009) (see above), including *D. globulatus* n. sp. and *D. conchatus* n. sp. The presence of a three-armed ventral bar, together with the absence of a membranous skirt around the margin of the copulatory tube base, allows this species to be distinguished from *D. ergensi*, *D. globulatus* n. sp. and *D. conchatus* n. sp. *Dactylogyrus sagittarius* n. sp. differs from all the species of the ‘ergensi’ group by the shape of the dorsal anchor (i.e. an anchor having an elongate evenly curved shaft and well-differentiated but very short point).

**Fig. 6.**
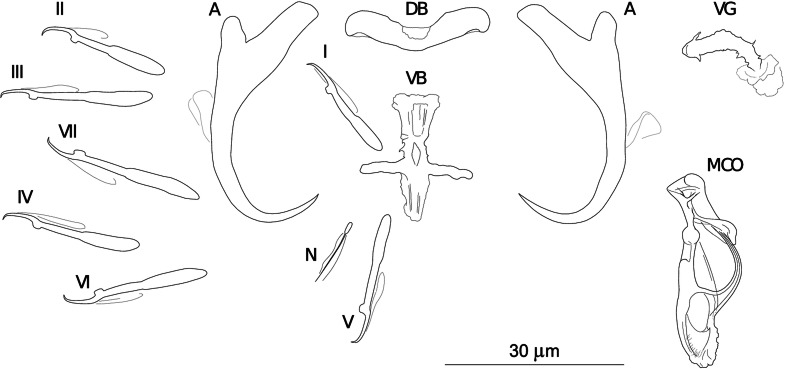
Sclerotized structures of *Dactylogyrus opertus n. sp. ex Telestes muticellus.* A, anchor; DB, dorsal bar; VB, ventral bar; N, needle; I–VII, hooks; VG, vagina; MCO, male copulatory organ.

***Dactylogyrus opertus* n. sp. (**Fig. 6**)**

urn:lsid:zoobank.org:act:0136688B-7547-436F-8CC7-B66D8473ECE2

**Type host and locality:** *Telestes muticellus* (Bonaparte, 1837); Torrente Cerfone, Intoppo (43°26′12.03″N, 11°58′33.00″E).

**Site on host:** Gill lamellae.

**Type and voucher material:** IPCAS M-756 (holotype; one paratype; one hologenophore voucher).

**Representative DNA sequences:** A nucleotide sequence of partial gene for 28S rRNA (790 bp long; MK434964) and nucleotide sequences representing a fragment (984 bp long; MK434944) including partial gene for 18S rRNA (487 bp), the ITS1 region (491 bp) and 5.8S (6 bp). No intraspecific variability was found (four specimens were analysed).

**Etymology:** The specific name is derived from Latin (*opertus* = hidden, secret) and refers to the distal part of the copulatory tube enclosed by (covered with) an accessory piece.

**Description** (based on four specimens): Body length 503 (499–507; *n* = 2); greatest width 77 (65–89; *n* = 2). Haptor with one pair of anchors (dorsal). Anchors with moderately developed roots forming acute angle (inner root longer); medially slightly constricted bent shaft; point; total length 38 (37–38; *n* = 4); inner root length 12 (11–13; *n* = 4); outer root length 4 (4–5; *n* = 4); point length 10 (9–10; *n* = 4). Anchor filaments well developed. Dorsal bar 25 (24–25; *n* = 4) wide, saddle-shaped. Ventral bar 19 (18–21; *n* = 4) wide, cross-shaped, with longitudinal aperture; anterior arm widened terminally; anterior and posterior arm each disrupted by elongate longitudinal splits. One pair of needles located near hooks of pair V. Hooks seven pairs; each with delicate point, depressed truncate thumb, shank comprised of two subunits (proximal subunit expanded); hook lengths: pair I 20 (19–21; *n* = 4); pairs II–VII 25 (22–29; *n* = 4). MCO composed of copulatory tube and basally articulated accessory piece; total length 33 (33–34; *n* = 4). Copulatory tube simple, evenly curved. Accessory piece with proximal part forming large flange around copulatory tube base; medial part composed of sclerotized supporting portion (terminating with spherical enlargement) and weakly sclerotized membrane (associated with copulatory tube base); terminal part bluntly finished, enlarged to form sheath directed backwards and enclosing the distal part of the copulatory tube. Vagina-like arrow with bent arrow shaft (shaft margins irregular); 16 (12–20; *n* = 4) long.

**Remarks:** *Dactylogyrus opertus* n. sp. was initially included in the study by Benovics *et al*. (2020) as *Dactylogyrus* sp. 7. *Dactylogyrus opertus* n. sp. was classified into the ‘nanus’ morphological group, showing a close resemblance to *D. nanus*, *D. nanoides* and *D. suecicus* (Pugachev *et al*., 2009) (see above). However, the new species differs from all species of the ‘nanus’ group mentioned above by possessing a cross-shaped ventral bar [the ventral bar is three-armed in *D. nanus*, *D. nanoides* and *D. suecicus* (the anterior arm divided into horn-like ends in *D. suecicus*)]. Further, the *D. opertus* n. sp. accessory piece of the MCO in *D. opertus* n. sp. is distinctive, having a bluntly finished terminal part, enlarged to form a sheath directed backwards and enclosing (covering) the distal part of the copulatory tube (in *D. nanus*, *D. nanoides* and *D. suecicus*, the distal part of the accessory piece is simply enlarged to form a sheath supporting the distal part of the copulatory tube in *D. nanus*, *D. nanoides* and *D. suecicus*).

The presence of a cross-shaped ventral bar makes *D. opertus* n. sp. similar to other species of the ‘nanus’ group, i.e. to *D. rutili* Glaeser, 1965 and *D. distinguendus* Nybelin, 1937. However, these two species, in contrast to the new species, belong to the species of the ‘nanus’ group having a proximally sharply curved copulatory tube and an elongate (S- or C-shaped) vagina (Pugachev *et al*., 2009). While in *D. opertus* n. sp., the posterior arm of the ventral bar is of similar length to (or longer than) the transverse arm in *D. opertus* n. sp., the ventral bar in *D. rutili* and *D. distinguendus* has a short posterior arm in *D. rutili* and *D. distinguendus* (i.e. a posterior arm shorter than the transverse arm; Pugachev *et al*., 2009). *Dactylogyrus opertus* n. sp. also resembles *D. sandai* Řehulková, Benovics et Šimková, 2020, a species recently described from Balkan cyprinoids and displaying close similarity with respect to the basic morphology of both the haptoral and reproductive structures (Řehulková *et al*., 2020). The anchors of the new species possess slightly more elongated points, and roots differ even more in their lengths. The anterior arm of the connective ventral bar in *D. opertus* n. sp. is also wider in comparison to *D. sandai.* Moreover, the terminal part of the MCO of *D. opertus* n. sp. forms a sheath pointing backwards and covering the distal part of the copulatory tube, while a similar structure in *D. sandai* does not enclose the copulatory tube but points forward.

### Phylogenetic position of the newly described *Dactylogyrus* species

**Fig. 7.**
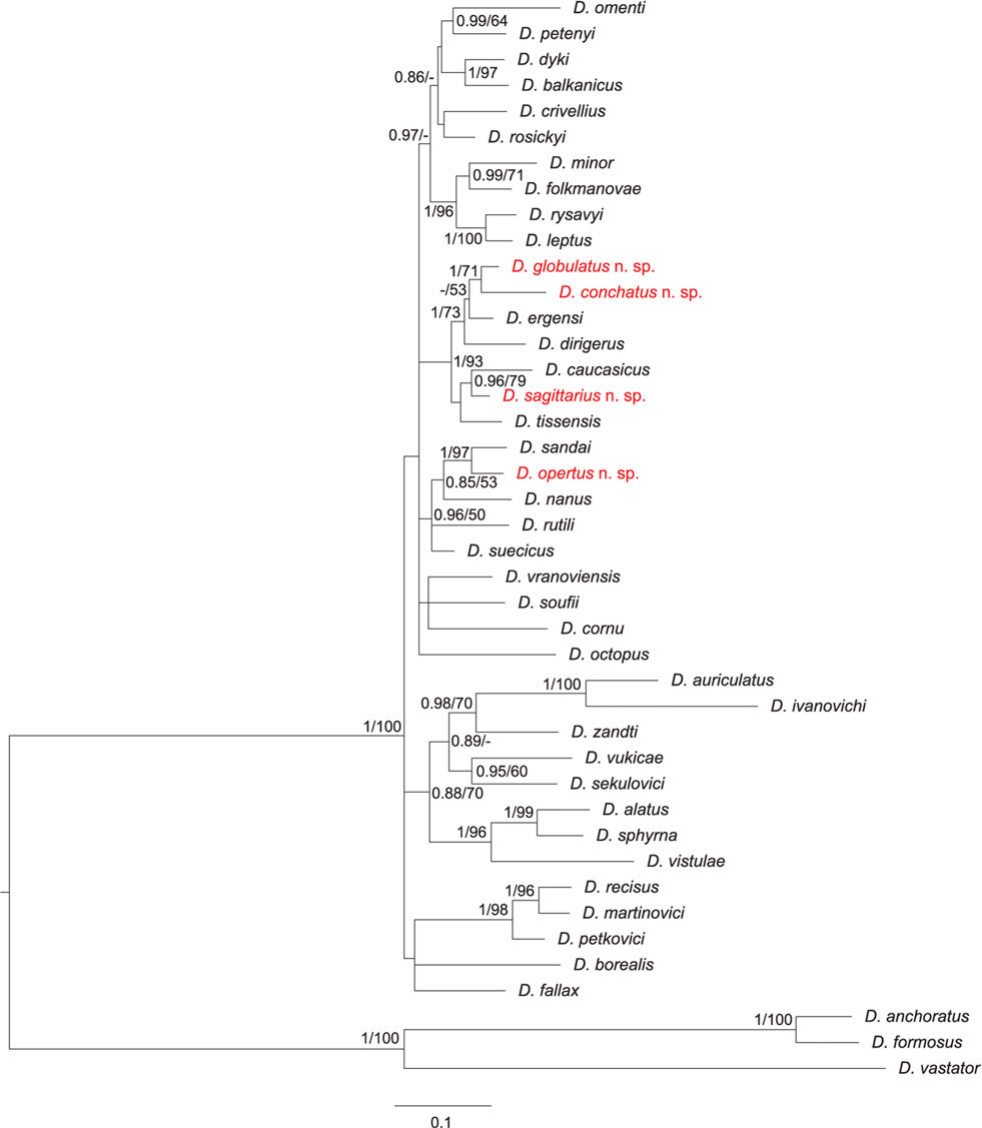
Phylogenetic tree of 41 *Dactylogyrus* species from European cyprinoids resulting from BI analysis. The tree is based on concatenated partial sequences of genes coding for 18S rRNA and 28S rRNA, and ITS1 region. Numbers along branches represent posterior probabilities (>0.80) and bootstrap support values (>50) for individual nodes, resulting from BI and ML analyses, respectively. Lower values are shown as dashes (*–*). Length of the branches represents the number of substitutions per site. Newly described *Dactylogyrus* species are shown in red colour.

The final concatenated alignment of the DNA sequences of the 42 *Dactylogyrus* species studied here spanned 1787 unambiguously aligned nucleotide positions (446 bp for 18S, 704 bp for 28S and 637 bp for ITS1). Both phylogenetic analyses (ML and BI) yielded trees with identical topologies, differing only in their node support values (see tree from BI in Fig. 7).

The phylogenetic analyses revealed that the newly-described *Dactylogyrus* species are associated with two phylogenetic lineages. *Dactylogyrus opertus* n. sp. is phylogenetically close to the recently described *D. sandai* – an endemic species of the Balkan Peninsula – and nested within a clade containing the morphologically similar species *D. nanus*, *D. rutili* and *D. suecicus*. All other new species, i.e. *D. conchatus* n. sp., *D. globulatus* n. sp. and *D. sagittarius* n. sp., are nested within the phylogenetic lineage that includes species with the ‘ergensi’ type of copulatory organ (i.e. *D. ergensi*, *D. dirigerus*, *D. caucasicus* and *D. tissensis*).

## Discussion

The species richness of a parasite community in fish usually reflects the effects of numerous abiotic factors related to the environment, the biology of the host and the diversity of the communities of syntopic organisms serving as intermediate hosts for many fish endoparasites (e.g. Gregory, 1990; Landsberg *et al*., 1998; Poulin, 2007). One of the less obvious factors is the introduction of non-native fish into a new region, as introduced fish may also serve as vectors for the co-introduction of non-native parasites (especially monogeneans, e.g. Havlátová *et al*., 2015; Benovics *et al*., 2017, 2018, 2020; Šimková *et al*., 2018; Kmentová *et al*., 2019; Wilson *et al*., 2019), which may represent a substantial threat to native host populations (Lymbery *et al*., 2014). Since current Italian freshwater fauna includes numerous non-native species (Bianco, 1995), it is tempting to expect that non-native parasites, co-introduced with their non-native host species, will also be present on endemic cyprinoids.

In the present study, we investigated ten species of endemic Italian cyprinoids from six sites in the northern part of the Apennine peninsula for their helminth parasites. The highest parasite diversity was observed among monogeneans, which are a group of ectoparasitic platyhelminths usually parasitizing either on the gills (*Gyrodactylus*, *Dactylogyrus* and *Paradiplozoon*), or on the skin (*Gyrodactylus*) of fish. A total of 20 *Dactylogyrus* species were identified, exceeding the number of species reported in the checklist of Galli *et al*. (2007). Herein, we reported 15 *Dactylogyrus* species that were not previously documented in Italy. Historically, Italian cyprinoids have not been comprehensively investigated for the presence of monogenean parasites. Before 2002, only eight monogenean species had been reported (Molnár and Ghittino, 1977; Bona *et al*., 1995). Some efforts to investigate the diversity of monogeneans came after the studies of Galli *et al*. (2002, 2005, 2007); however, endemic species were tackled only marginally, since studies were focused on investigating the parasite fauna of either invasive species [e.g. *C. gibelio* and *Cyprinus carpio* L.], or species with a wide distribution range across Europe [e.g. *Abramis brama*, *Squalius cephalus* (Linnaeus, 1758) or *Scardinius erythrophthalmus* (Linnaeus, 1758)]. The finding, in our study, of *D. achmerowi*, *D. falciformis* and *D. vastator* on endemic *B. plebejus* from the Po River is very unexpected [the presence of *D. vastator* on *B. plebejus* was also reported in the earlier work of Benovics *et al*. (2017)]. All three parasite species are host-specific to *C. carpio* and *C. gibelio* across their whole distribution range (e.g. Moravec, 2001; Galli *et al*., 2002; Jalali and Barzegar, 2005; Šimková *et al*., 2006, 2013; Ling *et al*., 2016). Their presence on *B. plebejus*, an endemic species of the Padano-Venetian ichthyogeographical district (*sensu* Bianco, 1990), may be a result of the introduction of the abovementioned alien species into the Apennines and the subsequent host-switch to endemic and phylogenetically close *Barbus* (all host species belonging to Cyprinidae). The presence of non-native *D. vastator* on the endemic Balkan *Aulopyge huegelii* Heckel, 1843 was previously reported by Benovics *et al*. (2017) and also explained by the host switch from *C. carpio* or *C. gibelio*. However, the alien *Dactylogyrus* species were not observed on *B. tyberinus*; therefore, we can postulate that host-switching occurs only in water bodies where congeneric *Barbus* lives in sympatry with introduced cyprinids, such as the Po River, where *C. carpio* and *C. gibelio* are commonly distributed (Galli *et al*., 2007; Kottelat and Freyhof, 2007).

**Fig. 8.**
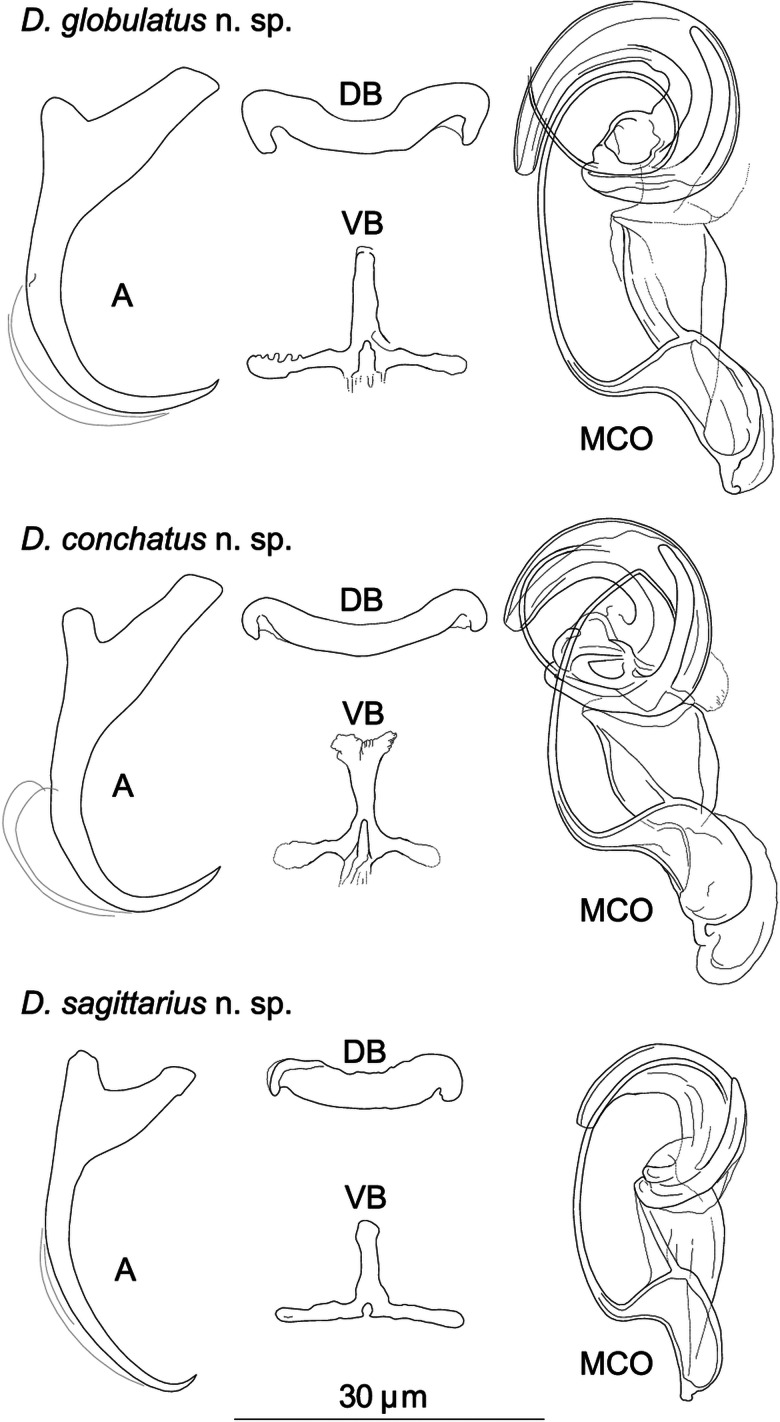
Sclerotized structures of three new *Dactylogyrus* species. A, anchor; DB, dorsal bar; VB, ventral bar; MCO, male copulatory organ.

The most common *Dactylogyrus* species on endemic cyprinoids was *D. vistulae* (present on 60% of investigated species), which is considered as a true generalist species with the widest host range in Europe (Moravec, 2001; Šimková *et al*., 2006; Benovics *et al*., 2018, 2020). Herein, we present *B. plebejus* as a new host of *D. vistulae*, increasing its host range to 34 cyprinoid species. In addition to already known *Dactylogyrus* species, we reported and described four new species from Apennine leuciscids. While *D. globulatus* n. sp., *D. opertus* n. sp. and *D. sagittarius* n. sp. appear to be host-specific to *C. soetta* (*D. globulatus* n. sp.) and *T. muticellus* (the latter two species), *D. conchatus* n. sp. was reported from two leuciscid species – *T. muticellus* and *P. genei*. The morphological similarity of *D. globulatus* n. sp., *D. conchatus* n. sp. and *D. sagittarius* n. sp. in the shape of their copulatory organs, and the fact that the only difference among them is in the shape of their haptoral elements suggest their common evolutionary origin (illustrations of the sclerotized structures directly taken from hologenophores are shown for comparison in Fig. 8). These endemic species most likely share a common ancestor with other species of the ‘ergensi’ morphological group, and evolved in the Apennines by subsequent intra-host duplication on *T. muticellus* (or its ancestor). The common origin of these three species and other species from the ‘ergensi’ morphological group (i.e. *D. caucasicus*, *D. dirigerus*, *D. ergensi* and *D. tissensis*) is also supported by our phylogenetic analyses. Endemic species most likely diverged and underwent speciation after the dispersion of their ancestor into the Apennine Peninsula. The differences in the haptoral elements might be adaptations for attaching to different substrates on different parts of the gill arch (i.e. microhabitats) of the same host species after intra-host speciation [morphological adaptations in the forms of monogenean haptors were suggested by Rohde (1979) and Šimková *et al*. (2000, 2002)]. A different evolutionary origin may be postulated for *D. opertus* n. sp., which more resembles, on the basis of morphology, species parasitizing on *Squalius*, e.g. *D. nanus*, *D. nanoides* and *D. suecicus* (see Pugachev *et al*., 2009 for morphotypes). *Dactylogyrus opertus* n. sp. [initially included as *Dactylogyrus* sp. 7 in the phylogenetic study by Benovics *et al*. (2020)] was found to be phylogenetically close to recently described *D. sandai* [identical with *Dactylogyrus* sp. 6 included in the study of Benovics *et al*. (2020)], a species from *Telestes* in the Balkans. The study of Benovics *et al*. (2020) also included a third species from *Telestes* (*Dactylogyrus* sp. 8 from *T. montenigrinus*), which has not yet been described; according to their study, all three *Dactylogyrus* species have a common evolutionary origin. Moreover, the present study confirmed the close morphological similarity between *D. sandai* and *D. opertus* n. sp. We can assume that these two species (probably also *Dactylogyrus* sp. 8 *–* a species of clade 6 in Fig. 1, Benovics *et al*., 2020) are genus-specific to *Telestes* (more specifically, they parasitize *T. karsticus* and *T. muticellus*, respectively). The two *Telestes* species are recently distributed over the Padano-Venetian ichthyogeographic district and, according to Buj *et al*. (2017), they share a common ancestor in the Balkans, which dispersed into the northern part of the peninsula and subsequently diverged into the Apennines. Therefore, we can assume that the evolutionary histories of host-specific *D. sandai* and *D. opertus* n. sp. are intimately linked with their *Telestes* hosts and that these *Dactylogyrus* species originated from cospeciation.

A total of 14 *Gyrodactylus* species were reported in this study, of which the most prevalent were *G. katharineri* and *G. laevis*. Both species exhibit a rather wide host range without obvious specificity to some hosts (each of them was reported from four Apennine cyprinoid species). They can be harboured by cyprinids, leuciscids and also gobiids (e.g. Gutiérrez-Galindo and Lacasa-Millán, 2001; Moravec, 2001; Harris *et al*., 2004; Djikanovic *et al*., 2012). *Gyrodactylus* cf. *markewitschi* was isolated from *Barbus caninus* from the Malezzo River. This species resembles *G. markewitschi*, which appears to be specific to the western Palearctic *Barbus* (Kakacheva-Avramova, 1973; Moravec, 2001; Harris *et al*., 2004), with some accidental infections reported from percids (Angelescu, 1974), or carp (Hussain *et al*., 2007). Therefore, *G.* cf. *markewitschi* might represent a species derived from *G. markewitschi*, endemic for the Apennine Peninsula. Unfortunately, to validate our hypothesis of the evolutionary proximity of these two species, molecular data for *G. markewitschi* would be required; however, these are still missing. The only locality where no *Gyrodactylus* parasites were recorded was Carmagnola, comprising gravel pits of relatively recent origin, filled with water from the Po River. Potential changes in abiotic factors from the river stream to the gravel pits (e.g. water current or temperature) may putatively have influenced the populations of gyrodactylid parasites and facilitated their reduction. For example, water temperature has a significant influence on parasite communities; therefore, the different water temperatures at the different sites in this study most probably influenced gyrodactylid communities to different degrees. Temperature directly affects the reproduction and survival time of *Gyrodactylus* (Jansen and Bakke, 1991; Bakke *et al*., 2007); during summer months, the intensity of gyrodactylids on hosts tends to decline (e.g. Koskivaara *et al*., 1991; Blažek *et al*., 2008). The water in gravel pits is captured from the Po River but, due to its stationariness, reaches a higher temperature than the running water of the River, which (considering studies above) might impact the parasite communities.

This study provides evidence that phylogenetically-close cyprinoid species from the same locality share generalist monogenean species. For example, all leuciscid species from Torrente Cerfone (i.e. *P. genei*, *R. rubilio*, *S. lucumonis*, *S. squalus* and *T. muticellus*) were parasitized by *D. vistulae.* Moreover, all abovementioned leuciscids, except *T. muticellus*, were also hosts of *P. megan*, suggesting that this diplozoid species does not exhibit host specificity in the investigated region, but rather a geographical specificity, which is in concordance with the previous reports of *P. megan* in cyprinoids (Benovics *et al*., 2021).

The fauna of intestinal helminths in the herein investigated cyprinoid species was species poorer in comparison to ectoparasitic monogeneans, and included only one species of eye fluke (metacercariae of *D. spathaceum*), three species of nematodes and one species of cestode. Among them, the most remarkable record is *B. rectangulum*, a tapeworm species from both investigated *Barbus* spp. from two geographically distant localities. While this representative of the monotypic genus *Bathybothrium* is relatively common in Barbinae distributed in the Palearctic region (Lühe, 1910; Joyeux and Baer, 1936; Protasova, 1977; Moravec and Amin, 1978; Van Maren, 1979; Dubinina, 1987; Scholz, 1989; Moravec, 2001), prior to this date there was no evidence of this cestode species in the Apennine Peninsula. The common host of *B. rectangulum* is *B. barbus* (e.g. Scholz and Moravec, 1996; Moravec, 2001), the *Barbus* species with the widest distribution range in Europe (Kottelat and Freyhof, 2007). *Bathybothrium rectangulum* reached relatively high prevalence and abundance in *B. barbus* (e.g. Laimgruber *et al*., 2005; Chunchukova and Kirin, 2018), and this widely distributed host species may have served as a potential vector for the distribution of *B. rectangulum* into different European regions. A more thorough ichthyoparasitological investigation in other southern European Peninsulas may potentially reveal other new hosts for this remarkable parasite species.

## Conclusions

Even though the Apennine Peninsula represents a region of high interest regarding ichthyological research, from the parasitological point of view, it remains underexplored, especially with respect to the parasites of endemic fish species. After more than a decade, we present the first study focused on the parasite fauna of endemic cyprinoids in northern Italy, and described four new monogenean species. We also found that local parasite communities appear to be influenced by the continual introduction of non-native fish into the region, potentially threatening native endemic species that are already undergoing reductions in their population sizes.

## Data Availability

The data supporting the conclusions of this study are included in this article. The type-material of the new species described in this study was deposited in the Helminthological Collection of the Institute of Parasitology, Czech Academy of Sciences, České Budĕjovice, Czech Republic under the accession number IPCAS M-753–M-756. The newly generated sequences were submitted to the GenBank database under accession numbers MW443031–MW443037.

## Supplementary material

For supplementary material accompanying this paper visit https://doi.org/10.1017/S0031182021000615. click here to view supplementary material
