# Supplementary material for: Helminth communities of endemic cyprinoids of the Apennine Peninsula, with remarks on ectoparasitic monogeneans, and a description of four new Dactylogyrus Diesing, 1850 species
Source: Parasitology. 2021 Apr 12;148(8):1003–18. doi: 10.1017/S0031182021000615 (PMC10090784; doi:10.1017/S0031182021000615)
Supplement: Supplementary file 1 [file S0031182021000615sup001.docx]

**Table S1.** *List of Dactylogyrus species used for phylogenetic analyses with GenBank accession numbers*

| *Dactylogyrus species* | Host species | Country of collection | 18S+ITS1 accession number | 28S accession number |
| --- | --- | --- | --- | --- |
| *Dactylogyrus alatus* | *Alburnus neretvae* | Croatia | MG792843 | MG792957 |
| *Dactylogyrus anchoratus* | *Carassius gibelio* | Croatia | KY859795 | KY863555 |
| *Dactylogyrus auriculatus* | *Abramis brama* | Czech republic | MG792838 | MG792952 |
| *Dactylogyrus balkanicus* | *Barbus prespensis* | Greece | KY201093 | KY201107 |
| *Dactylogyrus borealis* | *Phoxinus sp.* | Bosnia and Herzegovina | KY629343 | KY629372 |
| *Dactylogyrus caucasicus* | *Alburnoides devolii* | Albania | MG792840 | MG792954 |
| *Dactylogyrus conchatus n. sp.* | *Telestes muticellus* | Italy | MW443036* | MW443033* |
| *Dactylogyrus cornu* | *Vimba vimba* | Czech republic | KY629342 | KY629371 |
| *Dactylogyrus crivellius* | *Barbus prespensis* | Greece | KY201094 | KY201108 |
| *Dactylogyrus dirigerus* | *Chondrostoma vardarense* | Greece | MG792876 | MG792992 |
| *Dactylogyrus dyki* | *Barbus cyclolepis* | Greece | MG792856 | MG792971 |
| *Dactylogyrus ergensi* | *Chondrostoma vardarense* | Greece | MG792878 | MG792993 |
| *Dactylogyrus fallax* | *Vimba vimba* | Czech republic | KY629341 | KY629370 |
| *Dactylogyrus folkmanovae* | *Squalius cephalus* | Croatia | MG792911 | MG793028 |
| *Dactylogyrus formosus* | *Carassius gibelio* | Croatia | MG792869 | MG792984 |
| *Dactylogyrus globulatus n. sp.* | *Chondrostoma soetta* | Italy | MW443035* | MW443032* |
| *Dactylogyrus ivanovichi* | *Pachychilon pictum* | Greece | MG792883 | MG792999 |
| *Dactylogyrus leptus* | *Chondrostoma knerii* | Croatia | MG792871 | MG792986 |
| *Dactylogyrus martinovici* | *Pachychilon pictum* | Greece | MG792885 | MG793001 |
| *Dactylogyrus minor* | *Alburnus scoranza* | Albania | MG792848 | MG792962 |
| *Dactylogyrus nanus* | *Rutilus rubilio* | Italy | MK434933 | MK434953 |
| *Dactylogyrus octopus* | *Tropidophoxinellus spartiaticus* | Greece | MG792950 | MG793065 |
| *Dactylogyrus omenti* | *Aulopyge huegelii* | Bosnia and Herzegovina | KY201091 | KY201105 |
| *Dactylogyrus opertus n. np.* | *Telestes muticellus* | Italy | MK434944 | MK434964 |
| *Dactylogyrus petenyi* | *Barbus cyclolepis* | Greece | MG792857 | MG792972 |
| *Dactylogyrus petkovici* | *Pachychilon pictum* | Greece | MG792887 | MG793003 |
| *Dactylogyrus recisus* | *Pachychilon macedonicum* | Greece | MG792882 | MG792998 |
| *Dactylogyrus rosickyi* | *Pachychilon pictum* | Greece | MG792888 | MG793004 |
| *Dactylogyrus rutili* | *Rutilus lacustris* | Greece | MG792900 | MG793016 |
| *Dactylogyrus rysavyi* | *Alburnoides thessalicus* | Greece | MG792851 | MG792965 |
| *Dactylogyrus sagittarius n. sp.* | *Telestes muticellus* | Italy | MW443037* | MW443034* |
| *Dactylogyrus sandai* | *Telestes karsticus* | Croatia | MG792942 | MG793057 |
| *Dactylogyrus sekulovici* | *Pachychilon pictum* | Greece | MG792889 | MG793005 |
| *Dactylogyrus soufii* | *Telestes montenigrinus* | Albania | MG792946 | MG793061 |
| *Dactylogyrus sphyrna* | *Vimba vimba* | Czech republic | MG792951 | MG793066 |
| *Dactylogyrus suecicus* | *Rutilus lacustris* | Greece | MG792901 | MG793017 |
| *Dactylogyrus tissensis* | *Alburnoides thessalicus* | Greece | MG792852 | MG792966 |
| *Dactylogyrus vastator* | *Carassius gibelio* | Croatia | KY207446 | MW443031* |
| *Dactylogyrus vistulae* | *Squalis prespensis* | Greece | MG792925 | MG793043 |
| *Dactylogyrus vranoviensis* | *Squalius squalus* | Croatia | MG792931 | MG793048 |
| *Dactylogyrus vukicae* | *Delminichthys adspersus* | Bosnia and Herzegovina | MG792881 | MG792995 |
| *Dactylogyrus zandti* | *Abramis brama* | Czech republic | MG792839 | MG792953 |

Newly obtained sequences are marked by asterisks (*)
